# Predicting Mortality in Non-Variceal Upper Gastrointestinal Bleeding: Machine Learning Models Versus Conventional Clinical Risk Scores

**DOI:** 10.3390/jcm14207425

**Published:** 2025-10-21

**Authors:** İzzet Ustaalioğlu, Rohat Ak

**Affiliations:** 1 Department of Emergency Medicine, Gönen State Hospital, 10900 Balıkesir, Türkiye; 2 Department of Emergency Medicine, Dr. Lütfi Kırdar City Hospital, Health Sciences University, 34865 İstanbul, Türkiye; rohatakmd@gmail.com

**Keywords:** gastrointestinal hemorrhage, machine learning, mortality prediction, emergency medical services, clinical decision support

## Abstract

**Background/Objectives**: Non-variceal upper gastrointestinal bleeding (NVUGIB) is associated with considerable morbidity and mortality, particularly in emergency department (ED) settings. While traditional clinical scores such as the Glasgow-Blatchford Score (GBS), AIMS65, and Pre-Endoscopic Rockall are widely used for risk stratification, their accuracy in mortality prediction is limited. This study aimed to evaluate the performance of multiple supervised machine learning (ML) models in predicting 30-day all-cause mortality in NVUGIB and to compare these models with established risk scores. **Methods**: A retrospective cohort study was conducted on 1233 adult patients with NVUGIB who presented to the ED of a tertiary center between January 2022 and January 2025. Clinical and laboratory data were extracted from electronic records. Seven supervised ML algorithms—logistic regression, ridge regression, support vector machine, random forest, extreme gradient boosting (XGBoost), naïve Bayes, and artificial neural networks—were trained using six feature selection techniques generating 42 distinct models. Performance was assessed using AUROC, F1-score, sensitivity, specificity, and calibration metrics. Traditional scores (GBS, AIMS65, Rockall) were evaluated in parallel. **Results**: Among the cohort, 96 patients (7.8%) died within 30 days. The best-performing ML model (XGBoost with univariate feature selection) achieved an AUROC > 0.80 and F1-score of 0.909, significantly outperforming all traditional scores (highest AUROC: Rockall, 0.743; *p* < 0.001). ML models demonstrated higher sensitivity and specificity, with improved calibration. Key predictors consistently included age, comorbidities, hemodynamic parameters, and laboratory markers. The best-performing ML models demonstrated very high apparent AUROC values (up to 0.999 in internal analysis), substantially exceeding conventional scores. These results should be interpreted as apparent performance estimates, likely optimistic in the absence of external validation. **Conclusions**: While machine-learning models showed markedly higher apparent discrimination than conventional scores, these findings are based on a single-center retrospective dataset and require external multicenter validation before clinical implementation.

## 1. Introduction

Non-variceal upper gastrointestinal bleeding (NVUGIB) remains a critical clinical entity with substantial morbidity and mortality, particularly in emergency care settings. Despite advancements in endoscopic techniques and supportive care, NVUGIB continues to account for a significant proportion of acute gastrointestinal admissions and is associated with mortality rates ranging from 2% to 20% in various cohorts [1,2,3]. The heterogeneity of clinical presentations, coupled with diverse underlying etiologies, necessitates accurate early risk stratification to inform timely triage, resource allocation, and intervention planning.

To support clinical decision-making, several prognostic scoring systems have been developed, including the Glasgow-Blatchford Score (GBS), AIMS65, and the Pre-Endoscopic Rockall score. These tools aim to predict adverse outcomes such as mortality, need for transfusion, or urgent endoscopic therapy using a limited set of clinical and laboratory variables obtainable at presentation [4,5,6]. While GBS has been shown to effectively identify low-risk patients suitable for outpatient management, its predictive power for mortality or the need for endoscopic intervention remains modest. Similarly, although AIMS65 is simple to calculate and widely used, studies have questioned its generalizability across populations and clinical scenarios [7]. Limitations such as inter-observer variability, reliance on subjective parameters, and fixed threshold cut-offs further restrict the applicability of these scoring systems in dynamic emergency department (ED) environments [8,9].

Recent advances in data science have paved the way for the application of machine learning (ML) algorithms in clinical prognostication. ML models can process large volumes of structured clinical data, identify complex nonlinear interactions among variables, and update prediction functions without relying on rigid parametric assumptions [10]. In emergency medicine, such algorithms have demonstrated promising results in predicting short-term outcomes, including mortality, need for intensive care, and complications across various acute conditions [11,12]. In the context of NVUGIB, ML-based approaches offer the potential to surpass the limitations of traditional scores by integrating a broader set of predictors and adapting to the variability inherent in real-world clinical presentations [13].

The ED represents a high-stakes setting where early identification of patients at risk for adverse outcomes is essential. Implementation of robust, interpretable, and real-time prediction models may enhance clinical workflows and improve patient outcomes by enabling risk-adapted management strategies. However, despite the availability of several clinical scores, there remains no validated, accurate, and widely applicable tool for predicting short-term mortality in NVUGIB. This gap represents a major unmet need in emergency practice. Moreover, benchmarking ML algorithms against established clinical scores is necessary to assess their added value and guide their integration into routine care [11,14]. Therefore, the present study specifically addresses this gap by evaluating the performance of multiple supervised ML models, using only pre-endoscopic data available at ED presentation, and directly comparing them with traditional scores. By doing so, we aim to highlight both the potential benefits and the current limitations of ML-based approaches for early mortality risk assessment in NVUGIB.

This study aims to compare the performance of multiple supervised ML models with conventional clinical risk scores in predicting mortality among patients with NVUGIB presenting to the ED.

## 2. Materials and Methods

### 2.1. Study Design

This study was designed as a single-center, observational cohort analysis conducted at the ED of Kartal Dr. Lütfi Kırdar City Hospital. We retrospectively identified and analyzed all eligible patients over a 3-year period from 1 January 2022 through 1 January 2025. The study protocol was approved by the hospital’s Institutional Ethics Committee (Decision No: 2025/010.99/17/43, Date: 25 June 2025), which also granted a waiver of informed consent due to the retrospective design.

### 2.2. Study Population

The study included adult patients (≥18 years) who presented to the ED with signs of NVUGIB. NVUGIB was defined as bleeding originating proximal to the ligament of Treitz, typically manifesting as hematemesis or melena. Only cases classified as NVUGIB—based on clinical evaluation and endoscopic confirmation—were eligible. Patients with variceal bleeding, indeterminate bleeding source, incomplete diagnostic work-up, or missing demographic, laboratory, or 30-day outcome data were excluded.

### 2.3. Data Collection and Variable Definitions

Clinical, laboratory, and outcome data were retrospectively extracted from electronic health records and endoscopy reports. Recorded variables included demographics, comorbidities (e.g., chronic liver disease, chronic kidney disease, cardiovascular disease, active malignancy), presenting symptoms (e.g., hematemesis, melena, syncope), vital signs (systolic blood pressure, heart rate, respiratory rate, Glasgow Coma Scale), and laboratory results obtained at admission (hemoglobin, platelet count, creatinine, albumin, lactate, INR). Risk scores were calculated for each patient using initial clinical and laboratory parameters: Glasgow-Blatchford Score (GBS), Pre-Endoscopic Rockall Score, and AIMS65. Definitions for score components followed published criteria [6,7]. For all patients, upper endoscopy was performed within 24 h of ED presentation by gastroenterologists. From endoscopy reports, the following findings were extracted: the source of bleeding (e.g., peptic ulcer, Mallory-Weiss tear, malignancy), Forrest classification for ulcer bleeding (Ia–III), presence of active bleeding at the time of the procedure, and whether endoscopic hemostatic therapy was performed. These findings were used to confirm eligibility for NVUGIB and to report descriptive outcomes. However, endoscopic variables were not included in machine learning model development, which was based solely on data available at ED admission. All patients underwent upper endoscopy within 24 h of presentation. In-hospital management data included transfusion requirement, ICU admission, endoscopic or surgical interventions, and length of stay. All data were independently extracted by two trained research investigators using a standardized collection form. In cases of discrepancy, a third senior researcher adjudicated the final entry. This approach was implemented to minimize selection and classification bias, and to ensure consistency and accuracy of the dataset. Endoscopic variables (Forrest classification, stigmata of recent hemorrhage, presence of active bleeding) were recorded for descriptive reporting only but were deliberately excluded from model development, as the primary aim was pre-endoscopic risk prediction at ED presentation. Variables with >20% missingness were excluded a priori. For the remaining variables, missing data rates were <5%. Imputation was performed using multivariate imputation by chained equations (MICE): predictive mean matching for continuous variables, logistic regression for binary variables.

### 2.4. Outcome

The primary outcome of this study was 30-day all-cause mortality, defined as death from any cause occurring within 30 days after the index emergency department presentation for NVUGIB. Mortality status was ascertained through hospital electronic health records and cross-checked with the national death registry. Patients without documented 30-day follow-up were excluded from the analytic cohort. This outcome was chosen to reflect the study’s primary objective of developing a pre-endoscopic, admission-time prediction model relevant to emergency department decision-making.

### 2.5. Feature Selection and Machine Learning

To ensure comprehensive benchmarking and robust predictive performance, we implemented seven supervised machine learning algorithms: logistic regression, ridge regression, support vector machine, random forest, extreme gradient boosting, naïve Bayes, and artificial neural networks. These algorithms were selected based on their demonstrated efficacy in structured clinical prediction tasks, including mortality, intensive care unit admission, and hemorrhagic complications [11,15]. Tree-based ensemble methods such as RF and XGBoost have consistently shown high discriminative power in studies involving gastrointestinal bleeding and acute care populations [12]. Neural networks, particularly suited for capturing nonlinear relationships in high-dimensional clinical data, were included to complement linear and tree-based models. In this study, the neural network was configured as a multi-layer perceptron architecture, trained with dropout regularization and early stopping to mitigate overfitting [10,16]. Prior to model training, we applied six feature selection strategies to optimize the balance between parsimony and predictive accuracy. These included regularization-based methods such as the least absolute shrinkage and selection operator; model-specific techniques such as Boruta and recursive feature elimination employing logistic regression or random forest classifiers; and filter-based approaches including univariate statistical selection (*p* < 0.20) and mutual information ranking [17,18,19]. Additionally, a baseline model utilizing all available features without selection was included for comparison. This diverse set of feature selection methods allowed evaluation of models across a spectrum of interpretability and predictive performance (Supplementary Table S1).

### 2.6. Analysis

All statistical analyses were performed using R version 4.4.2 (R Foundation for Statistical Computing, Vienna, Austria). Continuous variables were summarized as mean ± standard deviation or median [interquartile range], depending on normality assessed via visual inspection, and compared using Student’s *t*-test or Mann–Whitney U test. Categorical variables were compared using the Chi-square test or Fisher’s exact test, as appropriate.

We developed machine learning models using seven classification algorithms: logistic regression, ridge regression, random forest, support vector machine (SVM), naive Bayes, extreme gradient boosting (XGBoost), and neural network. Each model was trained using a stratified 70/30 split of the dataset. Feature selection was performed using seven strategies: LASSO regression, Boruta, univariate filtering (*p* < 0.20), mutual information, recursive feature elimination (RFE) and inclusion of all features—resulting in 42 unique model–feature set combinations.

The neural network was implemented as a multi-layer perceptron using the keras package. It consisted of two hidden layers with 64 and 32 units, respectively, each using ReLU activation and dropout regularization (0.3 and 0.2). The output layer applied a sigmoid activation function for binary classification. Optimization was performed using the Adam algorithm (learning rate = 0.001) and binary cross-entropy loss. Training was conducted for 100 epochs with early stopping based on validation loss. The logistic regression model was implemented without regularization, while ridge regression applied L2 penalization with the optimal λ selected via 10-fold cross-validation using the cv.glmnet function. The random forest model consisted of 500 trees using default depth and mtry. The XGBoost model was configured with 100 boosting rounds, a learning rate of 0.1, and a maximum depth of 6. The support vector machine (SVM) used a radial basis function kernel, with cost and gamma tuned via grid search. The naive Bayes classifier assumed conditional independence between features and was implemented using the naiveBayes function from the e1071 package. All models were developed using standard R packages.

For XGBoost, the initial configuration (100 boosting rounds, depth = 6, learning rate = 0.1) was treated as a baseline. A grid search explored 50–500 boosting rounds, depth 3–10, and learning rates 0.01–0.3. The best combination was chosen via 5-fold cross-validation.

For the neural network, batch size was set to 32, the optimizer was Adam (β1 = 0.9, β2 = 0.999, decay = 1 × 10^−6^, learning rate = 0.001), and training was capped at 100 epochs with early stopping (patience = 10). Dropout rates were 0.3 and 0.2 for the two hidden layers.

Stopping criteria and hyperparameter ranges for all models are listed in Supplementary Table S1 to ensure reproducibility.

Model performance was evaluated using area under the receiver operating characteristic curve (AUROC), F1-score, sensitivity, specificity, positive likelihood ratio and negative likelihood ratio, all reported with 95% confidence intervals. AUROC confidence intervals were derived via 1000 bootstrap replicates. Pairwise DeLong tests were used to identify statistically non-inferior models within each feature selection group. Calibration was assessed using Brier scores, and model comparisons were performed using 1000 bootstrap iterations. Clinical scores (GBS, AIMS65, and Pre-Endoscopic Rockall) were evaluated using the same metrics for direct comparison.

## 3. Results

A total of 1595 patients presenting to the emergency department with signs of upper gastrointestinal bleeding were screened during the study period. Of these, 109 (6.8%) were excluded due to variceal bleeding, 191 (12.0%) due to lack of endoscopic confirmation, and 62 (3.9%) due to missing key clinical data. The final analytic cohort consisted of 1233 patients with confirmed NVUGIB and complete records, of whom 96 (7.8%) died within 30 days. Baseline characteristics and clinical findings are summarized in Table 1. Compared with survivors, deceased patients were older (mean age 70.6 vs. 58.6 years, *p* < 0.001), and had more frequent liver disease (21.9% vs. 11.1%, *p* = 0.005), chronic kidney disease (18.8% vs. 9.8%, *p* = 0.009), and malignancy (18.8% vs. 5.1%, *p* < 0.001). Syncope was also more common in the deceased group (29.2% vs. 15.5%, *p* = 0.001). They presented with lower systolic blood pressure (100 vs. 116 mmHg, *p* < 0.001), higher respiratory rate (22 vs. 14 breaths/min, *p* < 0.001), and lower Glasgow Coma Scale scores (12.2 ± 2.1 vs. 13.9 ± 1.4, *p* < 0.001). Endoscopic etiologies and Forrest classifications by mortality status are provided in Supplementary Tables S2 and S3.

Laboratory results, risk scores, and in-hospital management details are shown in Table 2. Deceased patients had lower hemoglobin (9.0 ± 2.0 vs. 10.6 ± 1.5 g/dL, *p* < 0.001), platelet (166.6 ± 55.3 vs. 195.3 ± 41.5 ×10^9^/L, *p* < 0.001), and albumin (3.1 ± 0.5 vs. 3.6 ± 0.4 g/dL, *p* < 0.001). Creatinine (2.2 ± 1.0 vs. 1.3 ± 0.5 mg/dL, *p* < 0.001) and lactate (3.2 ± 1.6 vs. 1.6 ± 1.0 mmol/L, *p* < 0.001) were higher. All three clinical scores were also significantly elevated in the deceased group, particularly AIMS65 (2.4 ± 1.5 vs. 0.9 ± 1.0, *p* < 0.001). Transfusion need (36.5% vs. 12.8%, *p* < 0.001), active bleeding at endoscopy (30.2% vs. 12.3%, *p* < 0.001), and ICU admission (44.8% vs. 14.6%, *p* < 0.001) were also more common. The median length of stay was 10 days [IQR 6–15] in the deceased group, compared to 5 days [IQR 4–7] in survivors (*p* < 0.001).

**Figure 1:**
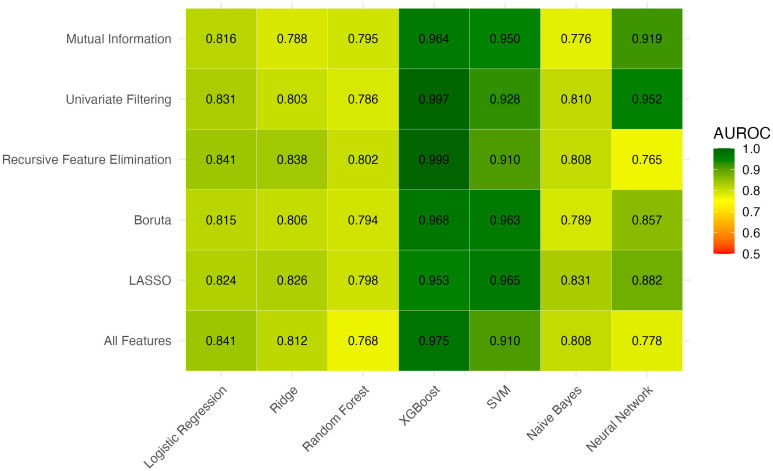
Area Under the Receiver Operating Characteristic Curve Values Across Machine Learning Models and Feature Selection Methods.

**Figure 2:**
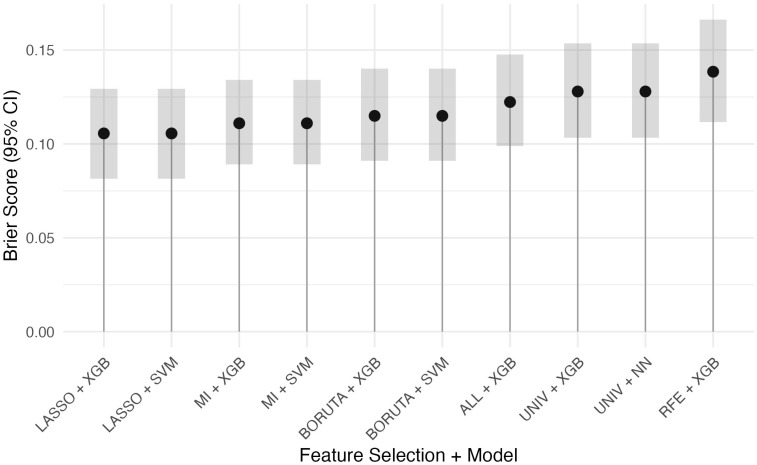
Brier Scores for the Best Performing Model(s) of Each Feature Selection Method. FS—Feature Selection; XGB—Extreme Gradient Boosting; SVM—Support Vector Machine; NN—Neural Network; MI—Mutual Information; UNIV—Univariate Selection; LASSO—Least Absolute Shrinkage and Selection Operator; RFE—Recursive Feature Elimination; ALL—All Variables (no feature selection applied).

A total of 6 feature selection strategies were applied and each was combined with 7 machine learning algorithms, resulting in 42 unique models. For each feature selection strategy, the model with the highest AUROC was identified, and models with statistically non-inferior AUROCs based on pairwise DeLong tests were retained (Figure 1). These top models were then compared by Brier score to assess calibration (Supplementary Table S4, Figure 2). Among the evaluated XGBoost models, white blood cell count and alanine aminotransferase consistently appeared as the top predictors based on SHAP values (Table 3).

Table 4 summarizes the discrimination and diagnostic performance of all candidate models and benchmark clinical scores. Among traditional tools, the Pre-Endoscopic Rockall Score had the highest AUROC (0.743) but remained inferior to all retained machine learning models (all *p* < 0.001 vs. top ML models). The best machine learning models also achieved higher sensitivity (e.g., Univariate + XGBoost: 83.3%) while maintaining excellent specificity (>99%). The highest F1-score was observed with Univariate + XGBoost (0.909; 95% CI 0.811–0.980).

## 4. Discussion

In this study, ML models significantly outperformed established clinical risk scores in predicting 30-day mortality for NVUGIB. The best-performing ML algorithms achieved AUROC values above 0.80, whereas the top conventional tool (the pre-endoscopic Rockall score) reached only 0.743 in our cohort. This finding is consistent with emerging evidence that data-driven models provide more accurate risk stratification in acute NVUGIB. For instance, Boros et al. reported that a gradient-boosted ensemble model attained an AUROC of ~0.84 for in-hospital GI bleed mortality, far exceeding the Glasgow-Blatchford Score (GBS, ~0.68) and Rockall (~0.62) on the same patients [2]. Similarly, Ungureanu et al. found that combining traditional scoring systems via ML improved mortality prediction beyond the capability of any individual score [20]. Likewise, in a critical care setting, an ML model significantly outperformed the APACHE IV severity index for predicting mortality among ICU patients with GI bleeding [21].

Several factors likely contribute to the superior performance of the ML models. The algorithms can integrate a broad range of clinical features (demographics, vital signs, laboratory values, comorbidities) and capture complex nonlinear relationships that fixed-form risk scores cannot. In our cohort, non-survivors had distinctly different profiles—older age, more comorbidities, hemodynamic instability (e.g., hypotension or tachycardia), and laboratory evidence of organ dysfunction (elevated creatinine, anemia, and hypoalbuminemia)—all of which an ML model can weigh appropriately. Traditional risk scores use only a handful of variables with predefined cut-offs, which may miss subtleties such as the magnitude of abnormalities or interactions between factors. In contrast, the ML approach is able to leverage these nuanced patterns in the data. Notably, one of our top models (an XGBoost classifier with feature selection) achieved an AUROC well above 0.80 with high sensitivity (~83%) while maintaining an excellent specificity (>99%). This represents a remarkable improvement over the best conventional score, the pre-endoscopic Rockall, and the difference in performance was highly significant. The ability of the ML model to attain high sensitivity and specificity simultaneously is particularly valuable in the emergency setting—it enables identification of high-risk patients without over-triaging those at low risk. While patients often present to the ED with undifferentiated UGIB, this study focused exclusively on non-variceal cases confirmed by endoscopy to ensure population homogeneity and reduce confounding from variceal etiologies. Importantly, all predictive variables used in the machine learning models were obtained prior to endoscopy. Therefore, the models are designed for pre-endoscopic risk stratification, similar to the GBS, AIMS65, and pre-endoscopic Rockall scores. We did not include the post-endoscopic Rockall score in the comparison, as it incorporates procedural findings not available at initial presentation.

A key obstacle to the clinical adoption of ML models is their limited interpretability. Complex architectures such as deep learning provide little transparency in decision-making, often being perceived as “black box” tools. To enhance clinical applicability, we explored variable importance using SHAP analysis, which consistently highlighted predictors such as white blood cell count, alanine aminotransferase, systolic blood pressure, blood urea nitrogen, lactate, and age. These features align with established clinical risk factors, lending face validity to the models. As the reviewer suggests, future work should consider translating these findings into a more interpretable format—such as a nomogram or simplified regression model based on the top predictors—to serve as a bridge between ML research and routine clinical practice.

Our results also illustrate how different ML strategies can be tuned to different clinical priorities. In our case, we prioritized overall accuracy and precision, as reflected by the XGBoost model’s very low false-positive rate. By comparison, some prior approaches favored maximizing sensitivity to ensure no critical case is missed. Shung et al. developed an ML model for NVUGIB that achieved ~100% sensitivity for adverse outcomes, but its specificity was only ~26% [12]. Such a high-sensitivity strategy guarantees that no high-risk patient is missed; however, it also flags many patients who ultimately have benign courses, potentially leading to unnecessary interventions and strain on resources. The optimal balance between sensitivity and specificity likely depends on the clinical context. For life-threatening bleeding, a higher false-positive rate may be acceptable, whereas in resource-limited settings a more specific approach might be preferable to avoid over-utilization of intensive care. Importantly, ML models output continuous risk probabilities, so the threshold for “high risk” can be adjusted as needed. This flexibility stands in contrast to traditional scores, which rely on fixed cut-offs that may not generalize well across different patient populations. We acknowledge that transparent reporting of ML model development is essential. To support independent replication, we provided full hyperparameter settings and training criteria in the revised Methods and Supplementary Table S1. Nonetheless, even with rigorous reporting, external replication on independent cohorts remains necessary before real-world application. Although endoscopic findings are strong prognostic indicators, they were excluded from our ML models because they are not available at the time of ED arrival, when triage and early management decisions are most critical. Including them would have shifted the analysis toward post-procedural rather than pre-endoscopic prediction.

Another insight from our analysis is the set of variables that consistently drove the ML predictions. Using various feature selection techniques, we found that several predictors repeatedly emerged as important, indicating that robust signals in the data were driving the models. Common top predictors included markers of hemodynamic instability (blood pressure, heart rate), organ dysfunction (creatinine, hemoglobin), and patient frailty/comorbidity (advanced age, chronic diseases). These align well with clinical intuition and prior NVUGIB studies, suggesting that the ML algorithms are recognizing genuine risk factors rather than spurious correlations. In fact, an interpretable ML study in critically ill NVUGIB patients identified a similar profile of key features—including shock index (combining heart rate and blood pressure), renal impairment, low albumin, advanced age, and altered mental status—as the most influential predictors of mortality [22]. This overlap implies that a relatively simple model focusing on these core variables might achieve nearly comparable accuracy. Such parsimony is advantageous for implementation; for example, a straightforward logistic regression using the top predictors could be explored if improved interpretability is needed without much loss in performance.

Hematemesis and hematochezia are key presenting symptoms in NVUGIB and may provide early clues about bleeding source and severity. In our study, hematemesis was more common among non-survivors, potentially indicating a higher risk of adverse outcomes; however, hematochezia was not systematically captured in our records. Further prospective studies are needed to evaluate the prognostic value of these symptoms, particularly in the context of machine learning–based risk stratification models.

It is also instructive to consider why the conventional risk scores underperformed in our cohort, as this highlights how risk assessment might be improved. GBS, AIMS65, and Rockall were all developed over a decade ago and each has known limitations. The GBS, while very sensitive for identifying patients who do not need intervention (indeed, a GBS of 0 correlates with extremely low risk) [23], was not designed to predict mortality and is less specific in stratifying the highest-risk cases. AIMS65 was intended to predict in-hospital mortality, and in our data AIMS65 scores were markedly higher in non-survivors, reflecting its inclusion of variables like shock and organ failure. However, AIMS65 uses coarse binary cut-offs (e.g., systolic BP < 90 mmHg or GCS < 14 as dichotomous criteria) and omits many other relevant factors; thus, its granularity and overall accuracy are modest. The pre-endoscopic Rockall score incorporates age and a measure of shock, which likely contributed to it performing best of the three traditional tools in our analysis. Yet Rockall too leaves out laboratory results and certain comorbidities, and it was meant only as an initial stratifier prior to endoscopy rather than a definitive mortality predictor. Furthermore, these one-size-fits-all scores may not account for important differences in specific patient populations. For example, NVUGIB in a patient with advanced liver disease carries unique risks (coagulopathy, portal hypertension, etc.) that generic scores might underweight. A recent study focusing on cirrhotic patients with NVUGIB found that a tailored ML model incorporating features such as coagulopathy and renal function outperformed conventional prognostic scores for that subgroup [24]. By using modern data-driven methods, our study essentially re-derived a risk prediction tool from the ground up, allowing the data to dictate which variables and thresholds are most predictive of mortality in the current era. The superior performance of the ML models suggests that risk stratification can indeed be refined beyond what legacy scores provide. Rather than abandoning traditional scores entirely, an intermediate step could be to develop an updated risk score or nomogram based on the ML model’s key predictors, which would serve as a more transparent bridge toward full ML integration. Ultimately, given the increasingly digital environment of healthcare, an ML-based approach—once validated—can be continuously updated and even tailored to specific hospital settings or patient subgroups, capabilities that static risk scores do not have.

Finally, although our focus was on mortality, the potential of ML models extends to other critical outcomes in NVUGIB. Many patients in our cohort required interventions such as blood transfusions, endoscopic therapy, or surgery; predicting these needs is also crucial for optimal triage and resource allocation. Prior studies have shown that advanced predictive models can forecast composite outcomes or the need for intervention with greater accuracy than standard scores. For instance, a recent study developed an ML classifier that identified patients requiring urgent hemostatic intervention with an AUROC of 0.84, significantly higher than the performance of the GBS (~0.75) [25]. Moreover, ML approaches have been used to predict complications like rebleeding with high accuracy [26], suggesting the opportunity for proactive management. It is conceivable that a single robust model could eventually provide a comprehensive risk profile for each patient on admission—estimating not only mortality risk but also the likelihood of rebleeding and the need for therapeutic intervention. This approach aligns with the direction of personalized medicine and could further streamline decision-making. For example, a patient deemed at high risk of rebleeding might undergo earlier repeat endoscopy or receive more aggressive proton pump inhibitor therapy. Our study lays the groundwork by demonstrating that ML can reliably stratify mortality risk; future research can expand these models to encompass other outcome dimensions in NVUGIB, ideally creating an integrated risk assessment platform for use at the bedside. Although several ML models achieved AUROC values approaching 1.0 in our internal dataset, these represent apparent discrimination and are unlikely to be reproducible in external populations. Such near-perfect results should be regarded with caution, as they almost certainly reflect optimism associated with retrospective single-center data. After accounting for this, we emphasize that ML models remain superior to conventional scores, but with expected real-world performance at more modest levels. It is important to acknowledge that the apparent superiority of ML models partly arises from their ability to incorporate a larger number of variables than conventional scores, which were intentionally designed to be simple and rapid to calculate at the bedside. Therefore, our results should not be interpreted as evidence that traditional scores are “flawed,” but rather that more detailed data, when available, can improve risk stratification. Future work could explore whether parsimonious ML models based on a limited subset of key predictors might achieve a balance between predictive performance and clinical practicality.

### Limitations

This study has several limitations. The single-center, retrospective design may limit the generalizability of our findings to other populations or care settings. Although we employed a stratified train-test split and applied 5-fold cross-validation within the training set to minimize optimism in performance estimates, all modeling was conducted on the same institutional dataset without external testing. As a result, the reported model metrics—particularly high AUROC and F1-scores—may reflect dataset-specific patterns rather than broader predictive validity. While every effort was made to reduce model complexity through feature selection and regularization, the potential for overly optimistic performance cannot be entirely excluded in the absence of external or prospective validation. Moreover, two predictors—length of stay and transfusion units—were recorded after the initial emergency department evaluation and thus may introduce temporal bias, although their SHAP values were low and unlikely to have driven performance. Additionally, because data were obtained from electronic health records, there may have been residual errors or missingness despite preprocessing. The relatively low event rate of 30-day mortality introduced a degree of class imbalance, which may have influenced discrimination and calibration metrics. Time-series data (e.g., serial vital signs or laboratory trends) were not included, which may have limited the temporal sensitivity of predictions. Finally, as the analysis was restricted to non-variceal bleeding, the results are not directly applicable to patients with variceal hemorrhage, and real-world deployment will require evaluation of model usability and integration into clinical decision-making processes. Excluding patients who did not undergo endoscopy may have introduced selection bias. These patients could include both low-risk individuals discharged early and high-risk individuals with rapid clinical deterioration or limitations of care, thereby potentially affecting the generalizability of the results. In addition, imaging modalities such as CT or upper GI series were not incorporated and may offer supplementary diagnostic or prognostic value, which could be explored in future prospective studies. The extremely high AUROCs reported here reflect apparent performance and are probably optimistic. Without external validation, these results should not be interpreted as achievable real-world accuracy. Future multicenter and prospective evaluations are essential to determine generalizability. The comparison between ML models and traditional scores was not entirely balanced, as ML models used dozens of laboratory and vital sign inputs whereas GBS, AIMS65, and Rockall were designed to rely on a few easily accessible parameters. This difference in information content likely contributed to the performance gap and should be taken into account when interpreting our findings. Our models intentionally excluded endoscopic predictors such as Forrest classification and active bleeding, despite their prognostic value, to maintain a pre-endoscopic scope. This design choice ensures clinical applicability in the ED, but may underestimate maximal achievable predictive accuracy. Although missingness was low (<5% per variable) and addressed using multiple imputation, residual bias from imputation strategies cannot be entirely excluded. Although complex ML models achieved high apparent discrimination, their “black box” nature limits immediate bedside adoption. Further work is needed to develop interpretable, clinically friendly formats that retain predictive accuracy.

## 5. Conclusions

ML-based models provided markedly improved prediction of 30-day mortality in NVUGIB compared to traditional clinical risk scores. By leveraging a richer set of clinical variables and complex pattern-recognition capabilities, the ML algorithms achieved higher discriminative ability and better calibration, potentially enabling more accurate early identification of high-risk patients (who may need intensive care or urgent intervention) and more confidence in managing low-risk patients conservatively. Before such models can be adopted in routine care, further validation is essential. External multicenter studies should confirm their performance in diverse populations, and prospective trials are needed to verify that ML-guided stratification actually improves clinical outcomes or resource use.

## Figures and Tables

**Table 1:** Baseline Characteristics at Presentation.

Variable	Survivor (n = 1137)	Deceased (n = 96)	*p*
Demographics			
Age (years)	58.6 ± 15.0	70.6 ± 13.2	<0.001
Sex—Male, n (%)	739 (65.0%)	63 (65.6%)	0.999
**Comorbidities**			
Liver disease, n (%)	126 (11.1%)	21 (21.9%)	0.005
Cardiac failure, n (%)	210 (18.5%)	23 (24.0%)	0.221
Chronic kidney disease, n (%)	111 (9.8%)	18 (18.8%)	0.009
Malignancy, n (%)	58 (5.1%)	18 (18.8%)	<0.001
History of peptic ulcer, n (%)	238 (20.9%)	30 (31.2%)	0.028
**Symptoms & Medication History**			
Syncope, n (%)	176 (15.5%)	28 (29.2%)	0.001
Melena, n (%)	768 (67.5%)	64 (66.7%)	0.910
Hematemesis, n (%)	338 (29.7%)	38 (39.6%)	0.050
Antiplatelet use, n (%)	150 (13.2%)	26 (27.1%)	<0.001
NSAID use, n (%)	144 (12.7%)	34 (35.4%)	<0.001
**Vital Signs & Neurological Status**			
Systolic BP (mmHg)	116 [106–127]	100 [87–116]	<0.001
Diastolic BP (mmHg)	76 [66–87]	62 [48–79]	<0.001
Heart rate (bpm)	91 [83–100]	105 [91–115]	<0.001
Respiratory rate (bpm)	14 [12–19]	22 [16–25]	<0.001
SpO_2_ (%)	96 [95–97]	93 [89–96]	<0.001
Body temperature (°C)	36.5 ± 0.4	36.5 ± 0.6	0.870
Glasgow Coma Scale	13.9 ± 1.4	12.2 ± 2.1	<0.001

BP, blood pressure; SpO_2_, peripheral oxygen saturation; NSAID, non-steroidal anti-inflammatory drug. Values are presented as mean ± SD or median [IQR] for continuous variables, and number (percentage) for categorical variables.

**Table 2:** Laboratory Results, Risk Scores, and In-Hospital Management.

Variable	Survivor	Deceased	*p*	Mean Difference (95% CI)
Laboratory Parameters				
Hemoglobin	10.6 ± 1.5	9.0 ± 2.0	<0.001	−1.6 (−2.0 to −1.2)
Platelets	195.3 ± 41.5	166.6 ± 55.3	<0.001	−28.6 (−40.1 to −17.2)
Albumin	3.6 ± 0.4	3.1 ± 0.5	<0.001	−0.5 (−0.6 to −0.4)
Creatinine	1.3 ± 0.5	2.2 ± 1.0	<0.001	1.0 (0.7–1.2)
BUN	26 [19–33]	44 [24–57]	<0.001	—
INR	1 [1–1]	2 [1–2]	<0.001	—
AST	38 [24–53]	58 [37–78]	<0.001	—
ALT	32 [21–42]	52 [35–65]	<0.001	—
WBC	8 [6–9]	11 [8–14]	<0.001	—
Lactate	1.6 ± 1.0	3.2 ± 1.6	<0.001	1.6 (1.3–1.9)
Risk Scores				
Glasgow Blatchford Score	6.1 ± 2.7	10.9 ± 5.3	<0.001	4.8 (3.7–5.9)
AIMS65	0.9 ± 1.0	2.4 ± 1.5	<0.001	1.4 (1.1–1.7)
PER Score	1.5 ± 1.2	3.0 ± 1.6	<0.001	1.6 (1.2–1.9)
Management & Hospital Course				
Transfusion	145 (12.8%)	35 (36.5%)	<0.001	—
Transfusion units	1 [0–2]	2 [1–4]	<0.001	—
Active bleeding at endoscopy	140 (12.3%)	29 (30.2%)	<0.001	—
Endoscopy not performed	13 (1.1%)	5 (5.2%)	0.010	—
Need for second endoscopy	30 (2.6%)	6 (6.2%)	0.055	—
ICU admission	166 (14.6%)	43 (44.8%)	<0.001	—
Rebleeding during stay	87 (7.7%)	6 (6.2%)	0.840	—
Length of stay (days)	5 [4–7]	10 [6–15]	<0.001	—

ALT, alanine aminotransferase; AST, aspartate aminotransferase; BUN, blood urea nitrogen; CI, confidence interval; INR, international normalized ratio; PER, Pre-Endoscopic Rockall; WBC, white blood cell. Values are presented as mean ± SD or median [IQR] for continuous variables, and number (percentage) for categorical variables. Mean difference is only shown for continuous variables with *p* < 0.05.

**Table 3:** Top Predictive Features Across XGBoost Models Based on SHAP Values.

Feature	Appeared in Top 10 (n)	Mean SHAP Value	Total SHAP Value
White blood cell count	6	1.03	6.18
Alanine aminotransferase	6	1.00	5.99
Systolic blood pressure	4	0.51	2.04
Blood urea nitrogen	3	0.40	1.20
Age	4	0.39	1.57
Lactate	3	0.39	1.18
Platelet count	4	0.39	1.54
Diastolic blood pressure	3	0.38	1.15
Albumin	3	0.31	0.93
Respiratory rate	5	0.30	1.49
Heart rate	4	0.27	1.09
Aspartate aminotransferase	4	0.24	0.97
Creatinine	3	0.23	0.68
Length of hospital stay	3	0.22	0.65
Hemoglobin	2	0.25	0.50
Oxygen saturation	1	0.56	0.56
International normalized ratio	1	0.20	0.20
NSAID use	1	0.12	0.12

**Table 4:** Ranked performance of robust models by AUC and 95% confidence intervals.

Model/Score	AUROC (95% CI)	F1 (95% CI)	Sensitivity (95% CI)	Specificity (95% CI)	+LR (95% CI)	−LR (95% CI)
GBS	0.717 (0.592–0.842)	0.539 (0.360–0.704)	0.499 (0.320–0.676)	0.971 (0.951–0.988)	19.238 (8.690–40.386)	0.513 (0.321–0.698)
AIMS65	0.696 (0.580–0.812)	0.358 (0.219–0.488)	0.465 (0.286–0.657)	0.903 (0.871–0.932)	4.842 (2.672–7.881)	0.589 (0.397–0.804)
PER	0.743 (0.629–0.857)	0.470 (0.310–0.615)	0.502 (0.323–0.683)	0.947 (0.923–0.970)	9.806 (4.800–18.254)	0.533 (0.334–0.719)
All Features + XGBoost	0.990 (0.981–0.998)	0.667 (0.487–0.812)	0.533 (0.351–0.724)	0.994 (0.985–1.000)	90.670 (30.620–228.670)	0.470 (0.280–0.660)
LASSO + XGBoost	0.963 (0.936–0.990)	0.638 (0.450–0.784)	0.500 (0.316–0.679)	0.994 (0.985–1.000)	85.000 (28.930–214.920)	0.500 (0.320–0.690)
LASSO + SVM	0.971 (0.946–0.995)	0.622 (0.433–0.780)	0.467 (0.290–0.667)	0.997 (0.991–1.000)	158.670 (38.160–213.390)	0.530 (0.330–0.710)
BORUTA + XGBoost	0.987 (0.976–0.997)	0.652 (0.455–0.788)	0.500 (0.318–0.667)	0.997 (0.991–1.000)	170.000 (42.210–222.300)	0.500 (0.330–0.690)
BORUTA + SVM	0.986 (0.976–0.996)	0.421 (0.200–0.596)	0.267 (0.111–0.424)	NA ^1^	NA ^1^	0.730 (0.580–0.890)
RFE + XGBoost	0.998 (0.995–1.000)	0.836 (0.711–0.933)	0.767 (0.607–0.917)	0.994 (0.985–1.000)	130.330 (46.290–304.960)	0.230 (0.080–0.400)
Univariate + XGBoost	0.999 (0.998–1.000)	0.909 (0.811–0.980)	0.833 (0.682–0.960)	NA ^1^	NA ^1^	0.170 (0.040–0.320)
Univariate + NN	0.997 (0.992–1.000)	0.760 (0.611–0.878)	0.633 (0.458–0.808)	0.997 (0.991–1.000)	215.330 (57.280–269.850)	0.370 (0.190–0.540)
MI + XGBoost	0.982 (0.968–0.997)	0.622 (0.426–0.773)	0.467 (0.286–0.654)	0.997 (0.991–1.000)	158.670 (35.940–212.660)	0.530 (0.350–0.720)
MI + SVM	0.965 (0.931–0.999)	0.591 (0.400–0.754)	0.433 (0.258–0.622)	0.997 (0.991–1.000)	147.330 (36.010–196.250)	0.570 (0.380–0.750)

AUROC, area under the receiver operating characteristic curve; F1, F1-score; BS, Glasgow-Blatchford Score; AIMS65, Albumin, International normalized ratio, Mental status, Systolic blood pressure, Age ≥ 65; PER, Pre-Endoscopic Rockall score; LASSO, Least Absolute Shrinkage and Selection Operator; BORUTA, Boruta feature selection algorithm; RFE, Recursive Feature Elimination; MI, Mutual Information; SVM, support vector machine; NN, neural network; XGBoost, extreme gradient boosting; +LR, positive likelihood ratio; −LR, negative likelihood ratio. ^1^ Confidence interval could not be calculated due to lack of variability in bootstrap samples (e.g., specificity = 100% across all resamples).

## Data Availability

The data supporting the findings of this study are available upon reasonable request from the corresponding author. Due to privacy and ethical restrictions, the dataset cannot be publicly shared.

## Supplementary Materials

The following supporting information can be downloaded at: https://www.mdpi.com/article/10.3390/jcm14207425/s1, Table S1: Variables Selected by Each Feature Selection Method; Table S2: Endoscopic Etiology by 30-day Mortality; Table S3: Forrest Classification in Peptic Ulcer Patients by 30-Day Mortality; Table S4: Pairwise Comparison of Brier Scores Between Feature Selection–Model Combinations.

## Abbreviations

The following abbreviations are used in this manuscript:

AIMS65	Albumin, INR, Mental status, Systolic blood pressure, Age ≥ 65 score
AUROC	Area Under the Receiver Operating Characteristic Curve
ED	Emergency Department
GBS	Glasgow-Blatchford Score
ICU	Intensive Care Unit
INR	International Normalized Ratio
ML	Machine Learning
NVUGIB	Non-Variceal Upper Gastrointestinal Bleeding
RFE	Recursive Feature Elimination
ROC	Receiver Operating Characteristic
XGBoost	eXtreme Gradient Boosting
