# Supplementary material for: Predicting Mortality in Non-Variceal Upper Gastrointestinal Bleeding: Machine Learning Models Versus Conventional Clinical Risk Scores
Source: J Clin Med. 2025 Oct 21;14(20):7425. doi: 10.3390/jcm14207425 (PMC12565200; doi:10.3390/jcm14207425)
Supplement: Supplementary file 1 [file jcm-14-07425-s001.zip › jcm-3859959-supplementary.pdf]

Supplementary Table S1. Variables Selected by Each Feature Selection Method

**Variable**	**Univariate Analysis**	**LASSO Regression**	**Mutual Information**	**Boruta Algorithm**	**Recursive Feature Elimination**	**All Variables**
Age	Yes	Yes	Yes	Yes	Yes	Yes
Sex						Yes
Liver disease	Yes				Yes	Yes
Cardiac failure		Yes				Yes
Chronic kidney disease	Yes	Yes			Yes	Yes
Malignancy	Yes	Yes			Yes	Yes
History of PUD	Yes				Yes	Yes
Syncope	Yes					Yes
Melena					Yes	Yes
Hematemesis	Yes					Yes
Glasgow Coma Scale	Yes		Yes		Yes	Yes
Systolic BP	Yes	Yes	Yes	Yes	Yes	Yes
Diastolic BP	Yes		Yes	Yes		Yes
Heart rate	Yes	Yes	Yes	Yes	Yes	Yes
Respiratory rate	Yes	Yes	Yes	Yes		Yes
Oxygen saturation	Yes	Yes	Yes	Yes		Yes
Hemoglobin	Yes		Yes	Yes	Yes	Yes

Blood urea nitrogen	Yes		Yes	Yes	Yes	Yes
INR	Yes		Yes	Yes		Yes
AST	Yes	Yes	Yes	Yes	Yes	Yes
ALT	Yes	Yes	Yes	Yes		Yes
White blood cell count	Yes	Yes	Yes	Yes	Yes	Yes
Creatinine	Yes	Yes	Yes	Yes	Yes	Yes
Albumin	Yes	Yes	Yes	Yes		Yes
Platelet count	Yes		Yes	Yes	Yes	Yes
Serum lactate	Yes	Yes	Yes	Yes	Yes	Yes
Antiplatelet use	Yes	Yes				Yes
NSAID use	Yes	Yes	Yes		Yes	Yes
Endoscopy timing		Yes				Yes
Etiology						Yes
Active bleeding	Yes					Yes
Transfusion units	Yes	Yes	Yes			Yes
Length of stay	Yes		Yes	Yes		Yes

Supplementary Table S2. Endoscopic Etiology by 30-day Mortality

**Etiology**	**Survivor (n=1137)**	**Deceased (n=96)**
Peptic ulcer (gastric or duodenal)	763 (67.1%)	52 (54.2%)
Dieulafoy lesion	26 (2.3%)	3 (3.1%)
Malignancy	40 (3.5%)	8 (8.3%)
Angiodysplasia / vascular lesion	38 (3.3%)	7 (7.3%)
Mallory-Weiss tear	45 (4.0%)	0 (0.0%)
Unspecified bleeding / undetermined lesion	85 (7.5%)	3 (3.1%)
Erythematous gastropathy / minimal findings	36 (3.2%)	1 (1.0%)
Unknown / missing	104 (9.1%)	22 (22.9%)

Supplementary Table S3. Forrest Classification in Peptic Ulcer Patients by 30-Day Mortality

**Forrest Classification**	**Survivor (n=763)**	**Deceased (n=52)**
Ia	23 (3.0%)	4 (7.7%)
Ib	64 (8.4%)	0 (0.0%)
IIa	101 (13.2%)	6 (11.5%)
IIb	210 (27.5%)	15 (28.8%)
IIc	132 (17.3%)	13 (25.0%)
III	233 (30.5%)	14 (26.9%)

Supplementary Table S4. Pairwise Comparison of Brier Scores Between Feature Selection–Model Combinations

**Model A**	**Brier A (95% CI)**	**Model B**	**Brier B (95% CI)**	**ΔBrier (95% CI)**
All Variables + Extreme Gradient Boosting	0.122 (0.099–0.148)	LASSO Regression + Extreme Gradient Boosting	0.106 (0.082–0.129)	-0.0122 (-0.0214–-0.0046)
All Variables + Extreme Gradient Boosting	0.122 (0.099–0.148)	LASSO Regression + Support Vector Machine	0.106 (0.082–0.129)	-0.0122 (-0.0214–-0.0046)
All Variables + Extreme Gradient Boosting	0.122 (0.099–0.148)	Boruta Feature Selection + Extreme Gradient Boosting	0.115 (0.091–0.140)	-0.0026 (-0.0066–0.0016)
All Variables + Extreme Gradient Boosting	0.122 (0.099–0.148)	Boruta Feature Selection + Support Vector Machine	0.115 (0.091–0.140)	-0.0026 (-0.0066–0.0016)
All Variables + Extreme Gradient Boosting	0.122 (0.099–0.148)	Recursive Feature Elimination + Extreme Gradient Boosting	0.138 (0.112–0.166)	0.0109 (0.0056–0.0168)
All Variables + Extreme Gradient Boosting	0.122 (0.099–0.148)	Univariate Selection + Extreme Gradient Boosting	0.128 (0.103–0.154)	0.0085 (0.0049–0.0126)
All Variables + Extreme Gradient Boosting	0.122 (0.099–0.148)	Univariate Selection + Neural Network	0.128 (0.103–0.154)	0.0085 (0.0049–0.0126)
All Variables + Extreme Gradient Boosting	0.122 (0.099–0.148)	Mutual Information + Extreme Gradient Boosting	0.111 (0.089–0.134)	-0.0067 (-0.0113–-0.0026)
All Variables + Extreme Gradient Boosting	0.122 (0.099–0.148)	Mutual Information + Support Vector Machine	0.111 (0.089–0.134)	-0.0067 (-0.0113–-0.0026)

LASSO Regression + Extreme Gradient Boosting	0.106 (0.082–0.129)	Boruta Feature Selection + Extreme Gradient Boosting	0.115 (0.091–0.140)	0.0094 (0.0023–0.0173)
LASSO Regression + Extreme Gradient Boosting	0.106 (0.082–0.129)	Boruta Feature Selection + Support Vector Machine	0.115 (0.091–0.140)	0.0094 (0.0023–0.0173)
LASSO Regression + Support Vector Machine	0.106 (0.082–0.129)	Boruta Feature Selection + Extreme Gradient Boosting	0.115 (0.091–0.140)	0.0094 (0.0023–0.0173)
LASSO Regression + Support Vector Machine	0.106 (0.082–0.129)	Boruta Feature Selection + Support Vector Machine	0.115 (0.091–0.140)	0.0094 (0.0023–0.0173)
LASSO Regression + Extreme Gradient Boosting	0.106 (0.082–0.129)	Recursive Feature Elimination + Extreme Gradient Boosting	0.138 (0.112–0.166)	0.0229 (0.0123–0.0349)
LASSO Regression + Support Vector Machine	0.106 (0.082–0.129)	Recursive Feature Elimination + Extreme Gradient Boosting	0.138 (0.112–0.166)	0.0229 (0.0123–0.0349)
LASSO Regression + Extreme Gradient Boosting	0.106 (0.082–0.129)	Univariate Selection + Extreme Gradient Boosting	0.128 (0.103–0.154)	0.0208 (0.0118–0.0304)
LASSO Regression + Extreme Gradient Boosting	0.106 (0.082–0.129)	Univariate Selection + Neural Network	0.128 (0.103–0.154)	0.0208 (0.0118–0.0304)
LASSO Regression + Support Vector Machine	0.106 (0.082–0.129)	Univariate Selection + Extreme Gradient Boosting	0.128 (0.103–0.154)	0.0208 (0.0118–0.0304)
LASSO Regression + Support Vector Machine	0.106 (0.082–0.129)	Univariate Selection + Neural Network	0.128 (0.103–0.154)	0.0208 (0.0118–0.0304)
LASSO Regression + Extreme Gradient Boosting	0.106 (0.082–0.129)	Mutual Information + Extreme Gradient Boosting	0.111 (0.089–0.134)	0.0055 (-0.0015–0.0124)

LASSO Regression + Extreme Gradient Boosting	0.106 (0.082–0.129)	Mutual Information + Support Vector Machine	0.111 (0.089–0.134)	0.0055 (-0.0015–0.0124)
LASSO Regression + Support Vector Machine	0.106 (0.082–0.129)	Mutual Information + Extreme Gradient Boosting	0.111 (0.089–0.134)	0.0055 (-0.0015–0.0124)
LASSO Regression + Support Vector Machine	0.106 (0.082–0.129)	Mutual Information + Support Vector Machine	0.111 (0.089–0.134)	0.0055 (-0.0015–0.0124)
Boruta Feature Selection + Extreme Gradient Boosting	0.115 (0.091–0.140)	Recursive Feature Elimination + Extreme Gradient Boosting	0.138 (0.112–0.166)	0.0137 (0.0066–0.0216)
Boruta Feature Selection + Support Vector Machine	0.115 (0.091–0.140)	Recursive Feature Elimination + Extreme Gradient Boosting	0.138 (0.112–0.166)	0.0137 (0.0066–0.0216)
Boruta Feature Selection + Extreme Gradient Boosting	0.115 (0.091–0.140)	Univariate Selection + Extreme Gradient Boosting	0.128 (0.103–0.154)	0.0112 (0.0064–0.0172)
Boruta Feature Selection + Extreme Gradient Boosting	0.115 (0.091–0.140)	Univariate Selection + Neural Network	0.128 (0.103–0.154)	0.0112 (0.0064–0.0172)
Boruta Feature Selection + Support Vector Machine	0.115 (0.091–0.140)	Univariate Selection + Extreme Gradient Boosting	0.128 (0.103–0.154)	0.0112 (0.0064–0.0172)
Boruta Feature Selection + Support Vector Machine	0.115 (0.091–0.140)	Univariate Selection + Neural Network	0.128 (0.103–0.154)	0.0112 (0.0064–0.0172)
Boruta Feature Selection + Extreme Gradient Boosting	0.115 (0.091–0.140)	Mutual Information + Extreme Gradient Boosting	0.111 (0.089–0.134)	-0.0040 (-0.0086–-0.0002)
Boruta Feature Selection + Extreme Gradient Boosting	0.115 (0.091–0.140)	Mutual Information + Support Vector Machine	0.111 (0.089–0.134)	-0.0040 (-0.0086–-0.0002)

Boruta Feature Selection + Support Vector Machine	0.115 (0.091–0.140)	Mutual Information + Extreme Gradient Boosting	0.111 (0.089–0.134)	-0.0040 (-0.0086–-0.0002)
Boruta Feature Selection + Support Vector Machine	0.115 (0.091–0.140)	Mutual Information + Support Vector Machine	0.111 (0.089–0.134)	-0.0040 (-0.0086–-0.0002)
Recursive Feature Elimination + Extreme Gradient Boosting	0.138 (0.112–0.166)	Univariate Selection + Extreme Gradient Boosting	0.128 (0.103–0.154)	-0.0026 (-0.0063–0.0005)
Recursive Feature Elimination + Extreme Gradient Boosting	0.138 (0.112–0.166)	Univariate Selection + Neural Network	0.128 (0.103–0.154)	-0.0026 (-0.0063–0.0005)
Recursive Feature Elimination + Extreme Gradient Boosting	0.138 (0.112–0.166)	Mutual Information + Extreme Gradient Boosting	0.111 (0.089–0.134)	-0.0174 (-0.0262–-0.0094)
Recursive Feature Elimination + Extreme Gradient Boosting	0.138 (0.112–0.166)	Mutual Information + Support Vector Machine	0.111 (0.089–0.134)	-0.0174 (-0.0262–-0.0094)
Univariate Selection + Extreme Gradient Boosting	0.128 (0.103–0.154)	Mutual Information + Extreme Gradient Boosting	0.111 (0.089–0.134)	-0.0151 (-0.0218–-0.0088)
Univariate Selection + Extreme Gradient Boosting	0.128 (0.103–0.154)	Mutual Information + Support Vector Machine	0.111 (0.089–0.134)	-0.0151 (-0.0218–-0.0088)
Univariate Selection + Neural Network	0.128 (0.103–0.154)	Mutual Information + Extreme Gradient Boosting	0.111 (0.089–0.134)	-0.0151 (-0.0218–-0.0088)
Univariate Selection + Neural Network	0.128 (0.103–0.154)	Mutual Information + Support Vector Machine	0.111 (0.089–0.134)	-0.0151 (-0.0218–-0.0088)
